# DISCONTOOLS: a database to identify research gaps on vaccines, pharmaceuticals and diagnostics for the control of infectious diseases of animals

**DOI:** 10.1186/s12917-016-0931-1

**Published:** 2017-01-03

**Authors:** Declan O’Brien, Jim Scudamore, Johannes Charlier, Morgane Delavergne

**Affiliations:** 1 International Federation for Animal Health Europe, Avenue de Tervueren 168, box 8, 1150 Brussels, Belgium; 2 Institute of Infection and Global Health, University of Liverpool, Liverpool, UK; 3 Avia-GIS, Risschotlei 33, 2980 Zoersel, Belgium

**Keywords:** Prioritisation, Animal disease, Research needs, EU

## Abstract

**Background:**

The public and private sector in the EU spend around €800 million per year on animal health and welfare related research. An objective process to identify critical gaps in knowledge and available control tools should aid the prioritisation of research in order to speed up the development of new or improved diagnostics, vaccines and pharmaceuticals and reduce the burden of animal diseases.

**Method:**

Here, we describe the construction of a database based on expert consultation for 52 infectious diseases of animals.

**Results:**

For each disease, an expert group produced a disease and product analysis document that formed the basis for gap analysis and prioritisation. The prioritisation model was based on a closed scoring system, employing identical weights for six evaluation criteria (disease knowledge; impact on animal health and welfare; impact on public health; impact on wider society; impact on trade; control tools). The diseases were classified into three groups: epizootic diseases, food-producing animal complexes or zoonotic diseases.

**Discussion:**

The highly ranked diseases in the prioritisation model comprised mostly zoonotic and epizootic diseases with important gaps identified in vaccine development and pharmaceuticals, respectively. The most important outcome is the identification of key research needs by disease. The rankings and research needs by disease are provided on a public website (www.discontools.eu) which is currently being updated based on new expert consultations.

**Conclusion:**

As such, it can become a reference point for funders of research including the European Commission, member states, foundations, trusts along with private industry to prioritise research. This will deliver benefits in terms of animal health and welfare but also public health, societal benefits and a safe and secure food supply.

## Background

Animal diseases are estimated to reduce the production of animal products by at least 20% according to the World Organisation for Animal Health (OIE) [1]. As such, the prevention and control of animal diseases has benefits in terms of animal health and welfare but also human health where zoonoses are concerned and broad societal benefits in terms of companion animal health and the security of a safe food supply.

In terms of funding, it is estimated that the public sector spends €400 million per year in Europe on animal health and welfare related research [2, 3] and the private sector spends €400 million per year on animal health research [4]. With about €800 million being spent per year, the added value of an objective process to prioritise critical research can be appreciated. By focusing a proportion of this expenditure on critical gaps in priority diseases, it will be possible to speed up the development and delivery of new and improved disease control tools including diagnostics, vaccines and pharmaceuticals to reduce the burden of disease on animals. Given that the current value of animal based products at producer prices in the E.U. is €154 billion per year [5], every percentage reduction in the impact of animal disease on production would be of major economic importance.

During the work of the European Technology Platform for Global Animal Health (ETPGAH)^1^ from 2004 to 2012, it was recognised that disease prioritisation was one of the most important initiatives that needed to be undertaken to focus and prioritise research [6]. This work necessitates the identification of gaps in knowledge as well as control tools – diagnostics, vaccines and pharmaceuticals.

The DISCONTOOLS project was funded under the EU 7th framework programme from 2008 to 2013 and originated from the Action Plan of the ETPGAH. The general objective of the project was to evaluate global animal health priorities and the risk they could pose to the European Union. This understanding would assist in ensuring the most effective allocation of research funding. The project led to the development of a disease database containing a gap analysis and prioritisation model for 52 infectious diseases of animals. The objective of this paper is to describe the different steps in the development of the database (Section Construction and content), describe its utility (Section Utility) and discuss how it could assist policy makers in targeting research funding (Section Discussion). It should be noted that the focus of the database is on research needs with respect to control tools in the form of diagnostics, vaccines and pharmaceuticals. It does not necessarily consider disease control strategies or other aspects of disease control such as disease modelling, surveillance and regulatory support.

## Methods

### General approach

**Fig. 1:**
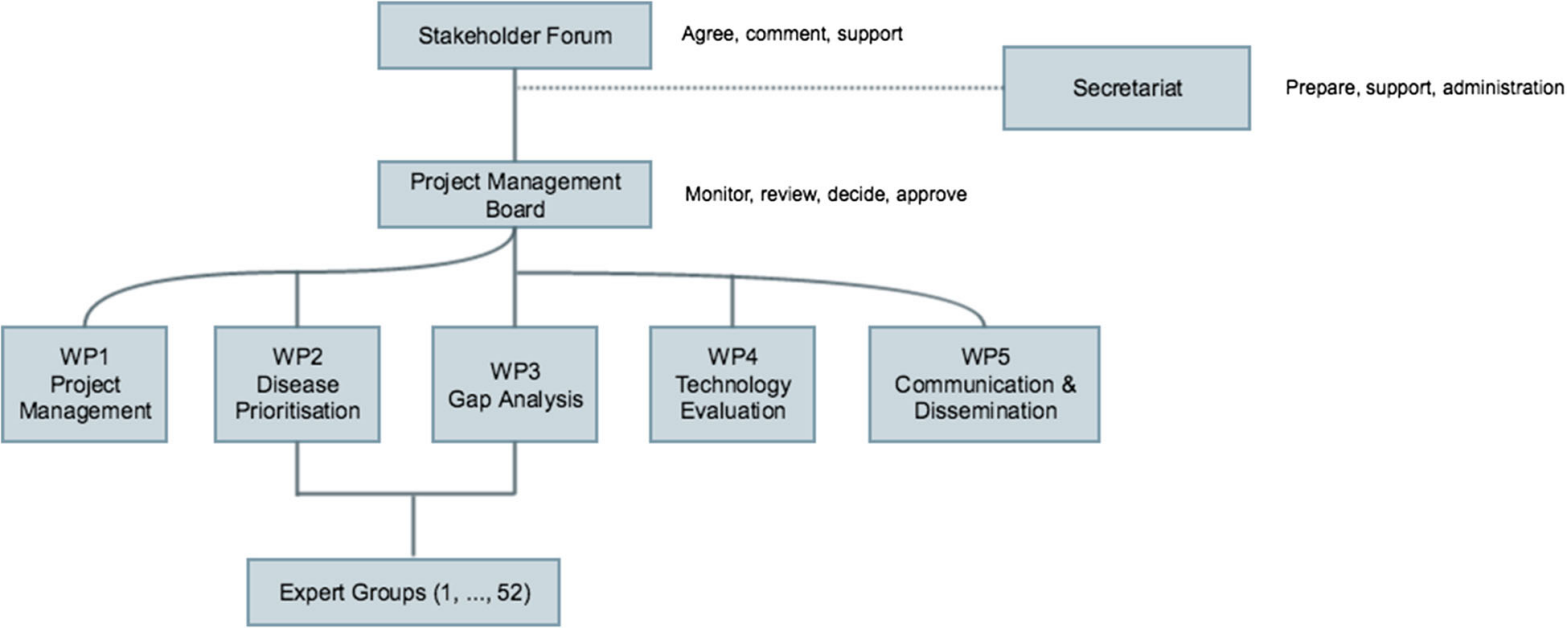
Organisation of the DISCONTOOLS project

The DISCONTOOLS project was organised as shown in Fig. 1. All European and Global organisations with an interest in animal health research were invited to join the Stakeholder Forum. It included organisations ranging from farmers, veterinarians and the pharmaceutical industry to chief veterinary officers, research institutes and related projects funded by the European Commission. The Project Management Board (PMB) comprised 10 representatives from the Stakeholders selected to represent research, industry, users and public bodies. The membership is listed in Table 1.

**Table 1:** Organisations represented in the Project Management Board of DISCONTOOLS

Organisation	Description
Copa-Cogeca	European union for agricultural organisations and cooperatives
CVO’s	Chief Veterinary Officers
DG Research	Directorate-General for Research and Innovation of the European Commission
EAEVE	European Association of Establishments for Veterinary Education
EMIDA ERA-net	Coordination platform of research on emerging infectious diseases of animals
EMVD	European Manufacturers of Veterinary Diagnostics
EPIZONE	International network of veterinary research institutes working on epizootic animal diseases
FVE	Federation of Veterinarians of Europe
HMA	Heads of Medicines Agencies
IFAH-Europe	International Federation for Animal Health Europe
MEDVETNET	European Network of Excellence for Zoonoses Research
OIE	World organisation for animal health

Five work packages or working groups were established each reporting to the PMB. Of these two working groups one on disease prioritisation, the other on gap analysis were involved in developing the database (See Fig. 1). Membership of the two working groups consisted of approximately 15 people and was by invitation along with nominations from the stakeholders and interested parties. It was important that each of the groups was balanced with members with appropriate expertise from research, industry, users (including farming and veterinary profession) and regulators as well as the European Commission and international organisations.

The development of the database was preceded by a review of existing processes for prioritisation and gap analysis by the PMB in order to steer the methodology adopted. Subsequently, the disease list was selected and prioritisation methodology developed, followed by expert opinion elicitation to provide content to the database. The prioritisation methodology was subdivided in the development of Disease & Product analysis document (D&P), a prioritisation model and gap analysis model.

### Review of existing prioritisation models

A worldwide review of existing models was carried out. In public health, a number of studies have methodically prioritised communicable diseases and pathogens [7–15]. In the field of animal health, most studies have focused on prioritisation of food-borne and zoonotic pathogens [13, 16–20]. In addition, studies have also been conducted with the specific aims of prioritising surveillance of wildlife pathogens [21], disease control for poverty alleviation [22], non-regulatory animal health issues [23] and exotic diseases and emerging animal health threats [24, 25].

Although methodological approaches differ, priority-setting studies typically follow a series of steps: (i) selecting a group of diseases/pathogens for prioritization; (ii) identifying a list of appropriate and measurable criteria to assess diseases/pathogens; (iii) defining a range of levels for each criterion; (iv) determining the relative importance by means of a weight or score for each level and criterion; (v) aggregating to produce an overall score for each disease/pathogen; and (vi) ranking diseases/pathogens by their overall score to derive a recommended list for prioritization [26]. It was evident that risk-based priority-setting should be systematic, flexible, reproducible and informative to public policy. The criteria must be explicit, measurable, relevant and objective wherever possible. However, the methodology and criteria required will depend on the goal of prioritisation, so a clear definition of the aim is essential. The priority-setting process should be transparent and open to discussion and revision. In addition, it is considered preferable to define a disease as specifically as possible (e.g. brucellosis of cattle versus brucellosis in general) and to consider how the model can evolve over time in order to remain of value. On the basis of the review, and considering the goal of DISCONTOOLS, i.e. identifying research needs of infectious animal diseases in the EU, the main steps which have been followed include establishing a list of relevant infectious diseases and gathering relevant information on each disease. It was considered essential that the scoring system allows diseases to be ranked based on total scores and/or on scores for particular criteria (e.g. impact on public health).

### Establishment of disease list

The working groups of the DISCONTOOLS project were created by inviting stakeholders to become involved in WP2, WP3 and/or WP4 with the PMB taking care of WP1 and WP5 (see Fig. 1). In parallel, the PMB invited an expert to Chair each of WP2, WP3 and WP4. The WPs then established a list of 52 priority diseases to be included in the prioritisation exercise. The starting point for the list of diseases was from the Action Plan of the ETPGAH which referred to 47 diseases. In addition, it was considered important not to lose sight of endemic diseases or disease syndromes (e.g. internal parasites, mastitis). Therefore, 3 groups of diseases were defined as follows:

1. epizootic diseases: infectious diseases which pose a risk for introduction or spread in the EU and for which tools for optimum detection, surveillance and control would be beneficial;
2. food producing animal complexes: major enzootic diseases of livestock in Europe;
3. zoonotic diseases: infectious diseases of animals that are important for human health and for their socio-economic effects.

The geographic dimension of the project was primarily European. Naturally, where a disease was not present in Europe, a global perspective was taken into account. The expert groups were asked to highlight and take into account strains and species where the economic impact was the highest not only in Europe, but worldwide.

After discussions of the working groups of DISCONTOOLS a final list of 52 diseases was agreed. The list was not considered to be exhaustive, but representative of most disease scenarios. Infectious diseases of aquatic animals or companion animals without a zoonotic implication were not considered.

## Results

### Prioritisation methodology

#### Disease and product analysis document

A D&P was developed for each disease by the working groups of the DISCONTOOLS project in order to have key information available prior to scoring of the different criteria. The D&P is a reference document that provides the detailed and relevant information for each disease which is necessary to support the scoring for the prioritisation and gap analysis models. It contains 23 main sections with sub-headings covering a wide range of aspects such as description and characteristics of the disease, route of transmission, zoonotic potential, control tools available, and socio-economic impact. The full list of sections may be consulted at www.discontools.eu/diseases under the custom report section. For each section, an additional column headed “Gaps identified” was included in the D&P to gather further information on the gaps in knowledge and products of each disease. The document was completed by the expert groups who were asked to reach a consensus on the final text which was then reviewed by the PMB.

#### Prioritisation model

The criteria, levels within each of the criteria, scores and weighting coefficients that were used in the prioritisation model can be viewed in Table 2. Six criteria were considered:

1. disease knowledge
2. impact on animal health and welfare
3. impact on public health
4. impact on wider society
5. impact on trade
6. control tools

**Table 2:** The prioritisation model: criteria considered, levels within the criteria, scores and applied weighting coefficients (Coef)

Criteria	Scores					Coef	Total (score*coef)
Disease knowledge	0	1	2	3	4	2.5	/100
1. Speed of spread							
2. Number of species involved							
3. Persistence of infectious agent in the environment							
4. Risk of spread to susceptible populations							
5. Potential for silent spread							
6. Wildlife reservoir and potential spread							
7. Vector reservoir and potential spread							
8. Variability of the agent							
9. Understanding of fundamental immunology							
10. Host pathogen interaction							
Impact on animal health and welfare	0	1	2	3	4	8.33	/100
1. Disease impact on production							
2. Duration of animal welfare impact							
3. Proportion of animals affected and suffering pain/injury/distress as a result of the disease							
Impact on public health – human health	0	1	2	3	4	4.16	/100
1. Impact of occurrence on human health							
2. Likelihood of occurrence							
3. Impact of occurrence on food safety							
4. Transmissibility (spread from animals to humans)							
5. Spread in humans							
6. Bioterrorism potential							
Impact on wider society	0	1	2	3	4	8.33	/100
1. Economic direct impact (including cumulative cost, e.g. enzootic vs. epizootic)							
2. Economic indirect impact (social, market)							
3. Agriterrorism potential							
Impact on trade	0	1	2	3	4	6.25	/100
1. Impact on international trade due to existing regulations							
2. Impact on EC trade due to existing regulations							
3. Potential for regionalisation							
4. Impact on security of food supply							
Control tools	+2	+1	0	−1	−2	16.66	/100
1. Appropriate diagnostics							
2. Appropriate vaccines							
3. Appropriate pharmaceuticals							
Total Score							

An interpretation guide (http://www.discontools.eu/upl/1/default/doc/1233_PrioInterV3-1-20110303.pdf) was developed to help the expert groups decide on the appropriate scores to apply to each criterion. The expert groups were asked to reach a consensus for the scoring of each criterion. A 5 – tiered scoring system was chosen as this appeared to offer a greater flexibility across the various criteria. The scoring scale applied to the 5-tiered system is as follows: for the first five sections 0, +1; +2; +3; +4 are used; for the sixth section dealing with control tool scores of +2; +1; 0; −1; −2 are used. This scoring scale was selected to highlight the differences in control tools for each disease in the sense that if for a particular disease a vaccine exists that has a high level of efficacy, quality, safety and availability, then a negative score will be attributed to the final total score of the concerned disease to diminish its priority as an effective tool is available. On the contrary, if control tools are missing, then a positive score will be added to the total score meaning that the disease will be higher in the prioritised list of diseases.

The weighting coefficient for each level of a criterion was computed as follows:

$$ W = \frac{100}{X*I} $$

where *W* = the weighting coefficient, *X* is the maximum score of a level within a criterion and *I* is the number of levels within a criterion. This ensured that the maximum score of each criterion was 100 and that the different criteria were attributed the same weight in the overall score.

As there are 6 criteria for each disease, the scores could be grouped, listed, ranked or presented in a wide range of ways using either the overall score or the individual scores for each criteria.

#### Gap analysis model of control tools

The criteria, levels within each of the criteria, scores and weighting coefficients that were used in the gap analysis model can be viewed in Table 3. Gap analysis considered 3 areas: diagnostic, vaccine and pharmaceutical gaps. The scoring system goes from +2 (important gap) to −2 (current tools are appropriate and no need to focus research in this area). As with the prioritisation model, an interpretation guide was developed to facilitate consistency in scoring (http://www.discontools.eu/upl/1/default/doc/1235_GapAna-Inter-V3-1.pdf).

**Table 3:** The gap analysis model: criteria considered, levels within the criteria, scores and applied weighting coefficients (Coef)

Criteria	Scores					Coef	Total (score*coef)
Diagnostic tools	2	1	0	−1	−2	4.17	/100
1. Availability^a^							
2. Prevention and control - Differentiation of infected from vaccinated (DIVA)							
3. Strategic reserve							
4. Capacity of production							
5. Market potential							
6. Affordable							
7. Quality/stability durability							
8. Sensitivity							
9. Specificity							
10. Reproducibility							
11. Simplicity/ease of use							
12. Speed							
Vaccination tools	2	1	0	−1	−2	4.55	/100
1. Commercial availability^a^							
2. Monitoring for infection in a vaccinated population							
3. Strategic reserve							
4. Capacity of production							
5. Market potential							
6. Affordable							
7. Quality/stability							
8. Safety of vaccines							
9. Efficacy							
10. Immunity							
11. Convenience of use							
Pharmaceutical tools	2	1	0	−1	−2	4.55	/100
1. Availability^a^							
2. Prevention and control							
3. Strategic reserve							
4. Capacity of production							
5. Market potential							
6. Cost							
7. Quality							
8. Safety animal							
9. Safety consumer/user concerns							
10. Safety environment							
11. Resistance							
Total Score:							

^a^A maximum score of 20 was given to the whole criterion when there isn’t any product available (not even under development)

### Expert opinion elicitation

An expert group leader was appointed for each disease and was asked to engage other experts. Where possible each group was asked to include experts with laboratory and diagnostic expertise, an epidemiologist, an industry representative and an individual with economic/trade expertise. The leader was expected to organise a physical or e-meeting in order to provide the information as described below. The names of the experts are published on the DISCONTOOLS website.

The average number of experts per group was 7. For 43 of the 52 included diseases (83%) the expert groups involved at least 4 members considered to cover the requested expertise (diagnostic, epidemiology, industry, economics). However for 9 diseases (17%), ≤3 experts were included (i.e. contagious bovine pleuropneumonia, swine influenza virus, peste des petits ruminants, rift valley fever, liver fluke, bovine herpes virus type 1, leptospirosis, salmonella, Crimean congo haemorrhagic fever).

## Utility

### DISCONTOOLS website

The DISCONTOOLS website contains two main sections: (i) work group pages and (ii) the disease database. The work group pages contain relevant minutes, documents and presentations related to meetings of the project management board.

The disease database contains the full D&P, along with a 2-page summary to make it easy to interpret the outcome. All the available information can be filtered for specific diseases or specific sections of the analysis and the customized reports can be downloaded in.pdf or.xls format. There is also a tool to enable web site users to provide comments on the D&P to the DISCONTOOLS secretariat. The prioritisation model and gap analysis model can be consulted for one or more diseases simultaneously and the specific scores for individual levels of criteria can be consulted or downloaded through custom reports.

### Ranking of diseases by prioritisation model, disease category and gap analysis model

In Table 4, diseases are ranked based on the total score of the prioritisation model. The Table is very useful in terms of providing a ‘Big Picture’ view and shows that the top ranked diseases comprise mostly zoonotic diseases and epizootic (often exotic) diseases. This, in turn, helps to guide funders who are working in an international environment and with a broad remit in terms of priorities. In Table 5, the diseases were ranked within disease category. This provides an opportunity to identify priorities within different research domains. As such, funders with an interest in public health will look to the zoonoses ranking. In contrast, funders focusing on international trade will have a great interest in the epizootic diseases ranking and funders who are focusing on the efficiency of production, especially within individual countries, will have a great interest in the ranking of food producing animal complexes. The results of the gap analysis model can be used to obtain more details of the gaps in control tools. As an example, in Table 6 we provide the scores of the gap analysis model for diagnostics, vaccines and pharmaceuticals for the top 10 ranked diseases within each disease category. In general, this table highlights the major gap in pharmaceuticals for the epizootic diseases which is not surprising as the availability of antivirals for the majority of diseases is very limited or non-existent at present. In contrast, for the food producing animal complexes, the picture is more diverse with, dependent on the disease, remaining gaps in diagnostics and vaccines and less so in pharmaceutical development. For the zoonotic diseases, good diagnostics are generally available and the analysis highlights the need for research into vaccine development. It should be noted that, whilst the gap is identified, in many cases, it is unlikely that a pharmaceutical solution would be pursued, for example Bluetongue.

**Table 4:** Ranking of 52 infectious diseases of animals by the overall score of the prioritisation model

Disease	Overall score
Nipah virus	464
Peste des petits ruminants	385
African swine fever	373
Rift valley fever	365
Bovine tuberculosis	359
Foot and mouth disease	310
Non tse-tse transmitted animal trypanosomiasis	296
African horse sickness	294
Cryptosporidiosis	291
Salmonellosis	282
Contagious bovine pleuropneumonia	269
Leishmaniosis	262
Brucellosis	254
Leptospirosis	250
Classical swine fever	247
Lumpy skin disease	244
Bluetongue	241
Orthopox	237
Hepatitis E virus	237
Poultry coccidiosis	226
Paratuberculosis	223
Anthrax	220
Campylobacter	219
Sheep and goat pox virus	218
Q-fever	214
Rabies	212
Avian Influenza	209
Verocytotoxigenic *Escherichia coli*	209
Liver fluke	202
*Chlamydophila abortus*	197
Nematodes	193
Porcine circo virus type 2	183
Bovine viral diarrhoea virus	183
Bovine spongiform encephalopathy	180
Small ruminant mastitis	179
Varroa mite	177
*Staphylococcus aureus* mastitis	175
Theileria	174
*Mycoplasma bovis*	173
Echinococcosis	167
Swine influenza virus	162
Congo crimean haemorrhagic fever	162
Swine *Actinobacillus pleuropneumonia*	159
Bovine respiratory syncytial virus	153
Parapox	152
Swine mycoplasma	143
Cysticercosis	130
Swine vesicular disease	118
West Nile Virus	118
Bovine herpes virus type 1	107
Porcine reproductive and respiratory syndrome virus	107
Environmental/Streptococcal mastitis	83

**Table 5:** Ranking of 52 infectious diseases of animals by disease category based on the prioritisation model

1. Epizootic diseases	Score	2. Food producing animal complexes	Score	3. Zoonoses	Score
Peste des petits ruminants	385	Poultry coccidiosis	226	Nipah virus	464
African swine fever	373	Paratuberculosis	223	Bovine tuberculosis	359
Rift valley fever	365	Liver fluke	202	Non tse-tse transmitted animal trypanosomiasis	296
Foot and mouth disease	310	Nematodes	193	Cryptosporidiosis	291
African horse sickness	294	Porcine circo virus type 2	183	Salmonellosis	282
Contagious bovine pleuropneumonia	269	Bovine viral diarrhoea virus	183	Leishmaniosis	262
Classical swine fever	247	Small ruminant mastitis	179	Brucellosis	254
Lumpy skin disease	244	Varroa mite	177	Leptospirosis	250
Bluetongue	241	*Staphylococcus aureus* mastitis	175	Hepatitis E virus	237
Orthopox	237	Theileria	174	Anthrax	220
Sheep and goat pox virus	218	*Mycoplasma bovis*	173	Campylobacter	219
Avian Influenza	209	Swine influenza virus	162	Q-fever	214
Parapox	152	Swine *Actinobacillus pleuropneumonia*	159	Rabies	212
Swine vesicular disease	118	Bovine respiratory syncytial virus	153	Verocytotoxigenic *Escherichia coli*	209
West Nile Virus	118	Swine mycoplasma	143	*Chlamydophila abortus*	197
		Bovine herpes virus type 1	107	Bovine spongiform encephalopathy	180
		Porcine reproductive and respiratory syndrome virus	107	Echinococcosis	167
		Environmental/Streptococcal mastitis	83	Congo crimean haemorrhagic fever	162
				Cysticercosis	130

**Table 6:** Scores from the gap analysis model for the top-10 ranked diseases within each disease category^a^

Disease	Diagnostics	Vaccines	Pharmaceuticals
1. Epizootic diseases			
Peste des petits ruminants	−5	−10	**20**
African swine fever	−50	**40**	**40**
Rift valley fever	**29**	**18**	**40**
Foot and mouth disease	−32	−20	**40**
African horse sickness	−18	**20**	**10**
Contagious bovine pleuro pneumonia	**9**	**30**	**25**
Classical swine fever	−23	−50	**40**
Lumpy skin disease	**27**	−5	**40**
Bluetongue	−27	−15	**40**
Orthopox	**18**	**40**	**15**
2. Food producing animal complexes			
Coccidiosis	**9**	**5**	−35
Paratuberculosis	0	0	**40**
Liver fluke	−9	**40**	−25
Nematodes	−5	**40**	−65
Bovine viral diarrhoea virus	−27	−10	**40**
Porcine circo virus type 2	**20**	−40	**40**
Small ruminant mastitis	−23	−5	−50
Varroa mite	**40**	**40**	−10
*Staphylococcus aureus* mastitis	**14**	0	−30
Theileria	**27**	**40**	−30
3. Zoonotic diseases			
Nipah virus	0	0	0
Bovine tuberculosis	−18	**10**	**40**
Non tse-tse transmitted trypanosomiasis	**32**	**40**	**15**
Cryptosporidiosis	0	**40**	−45
Salmonellosis	−23	−30	−35
Leishmaniosis	−14	**35**	−20
Brucellosis	−5	**20**	**40**
Leptospirosis	−5	**5**	−60
Hepatitis E virus	−32	**40**	**40**
Anthrax	**36**	**5**	−45

Positive scores (indicating a gap) were highlighted in bold

^a^Decimals were rounded to the first integer. This may cause apparent deviations between the sum of the individual and the total score

## Discussion

The aim of the DISCONTOOLS project was to build a prioritisation model and gap analysis on control tools as a means of prioritising research on infectious animal diseases with the support of stakeholders via a very open consultation process in the animal health research community.

The major achievement of this project consists of the wide and standardized consultation of the animal health research community, involving a total of 342 animal health scientists from all over the world. The establishment of expert groups was an important step in itself because it brought together scientists from different backgrounds and expertise often leading to lively debate, the challenge of assumptions and the identification of research gaps within their field. Where expert groups could not reach consensus, this was recorded in the D&P under the “Gap” section because it represents a gap in knowledge that needs to be filled by research.

An example is the benefit of bees to pollination. At the outset of the discussion, it was suggested by some of the participants that crop yields could decline by 65% without bees. However, when it was pointed out that wild insects have a more important role in pollination than honey bees, major crop species such as cereals are self-pollinating and many others are wind pollinated [27], it was agreed that the impact would be considerably less and it was noted that work needs to be done in this area to determine the real impact. Having had this discussion, it was recognised that efforts should be made to control *Varroa* mite as the bee sector – with its various products – is valuable (roughly estimated in the consortium at €640 million per annum) and needs to be protected.

As was done in Table 3, it is possible to rank diseases by total scores of the prioritisation model. The total score is valuable in highlighting the overall importance of some diseases. Nipah virus obtained the highest score. This was an interesting outcome and its high ranking is confirmed in other recent studies to prioritize diseases of food-producing animals and zoonoses [25, 28]. In the case of FMD, it scores highly due to knowledge gaps along with its impact on animal health and welfare, society and trade even though we have good diagnostics and vaccines. Given the impact of the disease, we need heat stable multi-valent vaccines and need to focus research in this area.

It might be surprising that the overall total for FMD (310) in the prioritisation model is lower than that for ASF (373) but an examination of the score in each of the six separate categories (Table 2) which make up the prioritisation model it is apparent where the variation exists. In the case of ASF the score is higher for the impact on international trade, animal health and welfare and significantly higher for control tools where there is no vaccine and diagnostic tests need to be improved.

Despite the maximally standardized consultation rounds and the use of a validation system in the form of Work Packages 2 and 3 and the PMB, the final ranking of diseases should be interpreted with caution. The total scores also hide specific research needs within a disease. In the case of nematodes for example, the total score hides the pressing need to assign resources to the development of vaccines in this area [29]. To avoid the user missing key data, the “Interpretation of the Scores Guide” was developed as well as the two page summary for each disease. The latter is of particular assistance to the non-specialist user who wants an overview of the critical research needs. Depending on the aim of the research, it may be more informative to compare specific criteria only between diseases (e.g. disease knowledge, impact on wider society, etc.). Two critical factors that can affect the results are: (i) the choice of the weighting coefficients and (ii) the different composition of each expert group. The different criteria of the prioritisation model such as disease knowledge, impact on animal health and welfare, etc. received an equal weight. However, depending on the user, different weight attributions may be desirable. Recently, novel methods have been developed to identify criteria important to different stakeholders by using the nominal group technique and attribute relative weights to the criteria using a wide consultation of different stakeholders and conjoint analysis [26]. This can result in different rankings according to the criteria and weights defined by different stakeholders (e.g. the public vs. health professionals) and provide additional insights to the decision maker on the spending of research funds [19]. In contrast to other prioritisation studies where all diseases were scored by the same expert panel, e.g. [13, 28], in the DISCONTOOLS project, each disease was scored by a different expert panel. This may have introduced bias due to inter-personal differences in scoring, but was considered necessary in order to adequately capture the current status of knowledge and gaps for control tools for each disease.

The epidemiological situation of infectious animal diseases can change rapidly (e.g. the current epidemic of the new porcine epidemic diarrhoea virus [30]), and biotechnological developments constantly change the landscape of diagnostics, vaccines and pharmaceuticals. As a consequence, the prioritisation exercise should be repeated regularly [28]. To this end, the database has been placed on a public website with the possibility that the public and research community can provide comments. This was deliberately done to provide an environment where the information in the D&P and the scores could be challenged. The idea is to gather comments over time and then ask the expert group to consider comments made and adjust the information and scoring on the site as appropriate. It is foreseen that the diseases will be systematically reviewed by the expert groups over a 5-year cycle, taking on board the latest technical advances. In fact, this process has already commenced and updated information on the site is available for African swine fever, Foot and mouth disease, Nematodes and Verocytotoxigenic *E. coli*.

## Conclusion

A database was established with the intention of identifying and prioritising research needs in the control of infectious animal diseases in the EU. If the focus of research is now placed on the priorities identified, it will hasten the development of diagnostics, vaccines and pharmaceuticals and reduce the 20% loss in production potential, valued at €28 billion per year in the EU. Funders of research including the European Commission, member states, foundations, trusts along with private industry should use the database to prioritise and focus future research. This will deliver benefits in terms of animal health and welfare but also public health, societal benefits and a safe and secure food supply. The employed method and results should not be considered fixed, but by refining the scoring methodology, challenging and updating the available information on a regular basis and incorporating new diseases, the DISCONTOOLS database has the potential to become a reference point used by stakeholders when prioritising research.

## Abbreviations

D&P: Disease and Product analysis document
ETPGAH: European Technology Platform for Global Animal Health
EU: European Union
OIE: World Organisation for Animal Health
PMB: Project Management Board

## Footnotes

1
www.etpgah.eu
